# Endemic fish calling: Acoustics and reproductive behaviour of the Neretva dwarf goby *Orsinigobius croaticus*

**DOI:** 10.1002/ece3.10673

**Published:** 2023-11-16

**Authors:** Sven Horvatić, Eric Parmentier, Stefano Malavasi, Maria P. Clara Amorim, Paulo J. Fonseca, Davor Zanella

**Affiliations:** ^1^ Department of Zoology, Faculty of Science University of Zagreb Zagreb Croatia; ^2^ Laboratory of Functional and Evolutionary Morphology, FOCUS University of Liège Liège Belgium; ^3^ Department of Environmental Sciences, Informatics and Statistics, Cà Foscari University of Venice Venezia Mestre Italy; ^4^ Departamento de Biologia Animal and MARE – Marine and Environmental Sciences Centre/ARNET ‐ Aquatic Research Network, Faculdade de Ciências Universidade de Lisboa Lisbon Portugal; ^5^ Departamento de Biologia Animal and cE3c ‐ Centre for Ecology, Evolution and Environmental Changes, Faculdade de Ciências Universidade de Lisboa Lisbon Portugal

**Keywords:** anatomical analysis, micro‐computed tomography, reproductive ethology, sound analysis

## Abstract

The Neretva dwarf goby *Orsinigobius croaticus* (Gobiiformes, Gobionellidae) is an endemic fish native to the freshwaters of the Adriatic Basin in Croatia and Bosnia and Herzegovina, a Mediterranean Biodiversity Hotspot. Due to its limited distribution range, specific karst habitat and endangered status, laboratory studies on reproductive biology are scarce but crucial. Herein, we investigated the sound production and acoustic behaviour of the endangered *O. croaticus* during reproductive intersexual laboratory encounters, utilising an interdisciplinary approach. We also performed dissections and micro‐computed tomography (μCT) scanning of the pectoral girdle to explore its potential involvement in sound production. Finally, comparative acoustic analysis was conducted on sounds produced by previously recorded soniferous sand gobies to investigate whether acoustic features are species‐specific. The endemic *O. croaticus* is a soniferous species. Males of this species emit pulsatile sounds composed of a variable number of short (~15 ms) consecutive pulses when interacting with females, usually during the pre‐spawning phase in the nest, but also during courtship outside the nest. Pulsatile sounds were low‐frequency and short pulse trains (~140 Hz, <1000 ms). Male visual behaviour rate was higher when co‐occurring with sounds and females entered the male's nest significantly more frequently when sounds were present. Characteristic body movements accompanied male sound production, such as head thrust and fin spreading. Furthermore, μCT scans and dissections suggest that *O. croaticus* shares certain anatomical similarities of the pectoral girdle (i.e. osseous elements and arrangement of *levator pectoralis* muscles) to previously studied sand gobies that could be involved in sound production. Multivariate comparisons, using sounds produced by eight soniferous European sand gobies, effectively distinguished soniferous (and sympatric) species based on their acoustic properties. However, the discrimination success decreased when temperature‐dependent features (sound duration and pulse repetition rate) were excluded from the analysis. Therefore, we suggest both spectral and temporal features are important for the acoustic differentiation of sand gobies.

## 1 INTRODUCTION

The Mediterranean Biodiversity Hotspot (MBH) is a widely renowned region for its significant environmental diversity and endangered wildlife (Darwall et al., 2014; Myers et al., 2000). The rivers of the Adriatic Sea Basin in Croatia are part of the MBH and Dalmatian freshwater ecoregions (Abell et al., 2008), and are especially rich in freshwater endemic fish, with 40 species or almost 30% of the total Croatian ichthyofauna, endemic to this area (Ćaleta et al., 2015, 2019; Kottelat & Freyhof, 2007; Myers et al., 2000). This endemism is a feature of the habitats of the Dinaric karst that covers roughly 54% of Croatian territory, with the presence of numerous caves, sinkholes, cold seasonal wells, and underground rivers (Kutle, 1999; Mrakovčić et al., 2006).

Among these endemic fish species, *Orsinigobius croaticus* (Mrakovčić et al., 1996), formerly described as *Knipowitschia croatica*, is a small benthic and short‐lived (less than 2 years) sand goby confined to the Dinaric karst of the Dalmatian ecoregion. It can be found exclusively in the freshwaters of Croatia and Bosnia and Herzegovina (Abell et al., 2008; Ćaleta et al., 2019; Horvatić et al., 2017; Tutman et al., 2020; Zanella et al., 2011). In Croatia, this species inhabits the Eastern part of the Adriatic Basin and has a naturally fragmented distribution range that includes the Neretva River, Matica River, the Vrgoračko Polje and Rastočko Polje fields and Baćina Lakes, some of which are NATURA 2000 sites (Ćaleta et al., 2015; Mrakovčić et al., 1996; Zanella et al., 2011, 2017; see Horvatić et al., 2017 for the map with its distribution area). In its natural habitat, *O. croaticus* occupies sandy bottoms with occasional stones/pebbles in karst rivers, slow‐flowing streams and oligotrophic lakes (Horvatić et al., 2017; Zanella et al., 2011). During winter and early spring, *O. croaticus* thrives in small rivers and streams whereas during the summer period, this goby survives in small karst underground ponds and refuges, when the watercourses completely dry out (Miller, 2004; Mrakovčić et al., 2006). On the IUCN Red List, *O. croaticus* is assessed globally as vulnerable (VU, B2ab(iii); D2, ver. 3.1.), but regionally as endangered (EN) due to its highly limited/fragmented habitat and declining habitat quality (Crivelli, 2018; Horvatić et al., 2017; Mrakovčić et al., 2006). However, this endangered status is also due to a lack of knowledge regarding its biological traits. There is little published data on the ecology or biology of *O. croaticus* (Horvatić et al., 2017; Mrakovčić et al., 2006; Zanella et al., 2011, 2017). Like other sand gobies, *O. croaticus* is a polygamous multiple spawner species that achieves sexual maturity quite early (i.e. within its first year), and reproduces from March to November, although most spawning occurs from April to September (Kottelat & Freyhof, 2007; Mazzoldi & Rassotto, 2001; Zanella et al., 2011, 2017).

The sand gobies are a monophyletic gobiiform group (Gobionellidae, Gobiiformes) of about 30 species in the genera *Knipowitschia*, *Pomatoschistus*, *Economidichthys*, *Ninnigobius* and *Orsinigobius* (Betancur‐R et al., 2017; Nelson et al., 2016; Thacker, 2009; Thacker et al., 2019; Tougard et al., 2021). They inhabit intertidal marine and coastal freshwater ecosystems with muddy‐to‐pebble bottom across Europe, including the waters of the Mediterranean, Ponto‐Caspian and Northeast Atlantic regions (Freyhof, 2011; Kovačić & Patzner, 2011; Miller, 2004; Šanda & Kovačić, 2009). Recent phylogenetic studies found evidence that sound production is widespread among actinopterygian fishes, suggesting that acoustic behaviour evolved independently multiple times in unrelated clades and that there is a strong selection for the use of sound production as a behavioural trait across vertebrate evolution (Fine & Parmentier, 2015; Rice et al., 2020, 2022). Communicative sound emission in fish is usually linked to courtship and spawning or aggressive behaviour (Amorim, 2006; Mann et al., 2008; Myrberg Jr. & Lugli, 2006). In fish bioacoustics, acoustic signals associated with reproductive intersexual interactions have been the most commonly studied types of sounds (Amorim, 2006), since it is believed that these sounds serve to attract potential mates (Longrie et al., 2013; Parmentier et al., 2010), to synchronise spawning activities at aggregation sites (Erisman & Rowell, 2017; Jublier et al., 2019; Lobel, 1992; Rowell et al., 2015) or to synchronise gamete release by conspecifics (Hawkins & Amorim, 2000; Lobel, 2002). Sand gobies are a common model group among soniferous actinopterygian fishes for sound production and have long been utilised in ethological and comparative bioacoustics studies. The acoustic abilities of sand gobies have been intensively investigated in the last 30 years, especially in the species of the genera *Pomatoschistus*, *Knipowitschia* and *Orsinigobius* (Amorim & Neves, 2007; Blom et al., 2016; Lugli et al., 1997; Malavasi et al., 2008, 2009; Parmentier et al., 2017; Torricelli et al., 1990; Zeyl et al., 2016). In eight sand goby species, either pulsatile or thump sounds (and sometimes both) have been recorded to date (Amorim & Neves, 2007; Blom et al., 2016; de Jong et al., 2016; Zeyl et al., 2016), while in *Economidichthys pygmaeus* (Holly, 1929) sounds were not detected during behavioural experiments (Gkenas et al., 2010). Most of our understanding regarding the acoustic abilities of Mediterranean sand gobies stems from the common, widely distributed and non‐threatened species assigned to the least concern (LC; IUCN Red List) category (Amorim et al., 2013; Blom et al., 2016; de Jong et al., 2016; Zeyl et al., 2016). However, since *O. croaticus* was regionally classified as a vulnerable species with a very restricted distribution (Crivelli, 2006, 2018; Horvatić et al., 2017), this research is the first study of the acoustic communication in endangered Mediterranean sand goby.

The main goal of this study was to investigate the acoustic communication of *O. croaticus* using an interdisciplinary approach. Specifically, our aims were to: (i) investigate the sound production of captive *O. croaticus* males and quantify acoustic parameters of the sounds; (ii) examine the reproductive behaviour of soniferous males and its association to sound production; (iii) provide insight into the putative sound‐producing mechanism by exploring the anatomy of the pectoral girdle and (iv) explore the acoustic diversification of soniferous sand gobies by quantitatively comparing acoustic signals between the study species and previously recorded Mediterranean sand gobies (genera *Ninnigobius*, *Pomatoschistus*, *Knipowitschia* and *Orsinigobius*).

## 2 MATERIALS AND METHODS

### 2.1 Fish sampling, laboratory housing and experimental design

*Orsinigobius croaticus* was caught using electrofishing (Hans Grassl, model: EL65 IIGI, power: 13 kW) from a boat during the spring 2019. Sampling was performed on the Matica River in Croatia (near the village Vina 43°10′30.33″ N, 17°23′12.36″ E). Direct current was used during sampling procedures since it causes galvanotaxis or an attraction zone where fish actively swim toward the anode, and is typically less harmful. All fish displaying electro‐tactic movement towards the anode or paralysis were sampled using dip nets. In total, we collected 25 individuals (15 males and 10 females) from the main river channel at a depth of 0.5–2 m. Fish were transferred alive to large plastic water containers equipped with aerators and transported to the laboratory at the Faculty of Science, University of Zagreb. At the laboratory, fish were sexed based on urogenital papilla and body colouration (Miller, 1986) and housed in four single‐sex community rectangular tanks (120 L; five females per tank; eight males per tank; Figure S1). Each community tank was equipped with 2–4 nests. After an acclimatisation period of 5–8 days, prospective soniferous males were chosen for subsequent laboratory acoustic‐visual recordings based on colouration (complete or partially darken body, fins and head; Zanella et al., 2011) and territoriality. Eight males ($\bar{x}$ ± SD = 49.21 ± 0.8 mm total length, *L*_T_; range: 48.07–50.06 mm; $\bar{x}$ ± SD = 41.07 ± 1.01 mm standard length, *L*_S_; range: 38.96–42.29 mm; $\bar{x}$ ± SD = 1.24 ± 0.11 g weight, *W*; range: 1.10–1.40 g) exhibiting typical reproductive behaviour were chosen for the experiments. Females (*N* = 5; 36.75 ± 5.10 mm *L*_S_; range: 28.75–41.27 mm) were chosen for the recording sessions according to yellow belly colouration, luminescent green spot on the first dorsal fin and dark eyes, all indicators of female readiness for spawning (Blom et al., 2016; Zanella et al., 2011).

We followed the acoustic‐visual recording protocol established by previous authors (Amorim et al., 2013; Amorim & Neves, 2007; Pedroso et al., 2013), where experimental tanks, placed on top of 3 cm thick rubber foam shock absorbers to reduce substrate‐born noise, were divided into three compartments separated by removable partitions (Figure S1). Each lateral compartment housed one territorial male with a nest (artificial tunnel‐shaped plastic cover, dimensions: length = 100 mm, width = 60 mm, height = 50 mm), while the middle section (‘arena’) was occupied by a ripe female. The female compartment was not provided with a nest. The eight males were divided into separate lateral compartments, where they remained throughout the experiments. Experimental fish were kept at natural photoperiod and fed daily ad libitum with *Daphnia*. Water temperature, monitored with a thermometer (Aquaterra) and manually regulated with a heater (Mylivell), was maintained between 18 and 22°C (natural range). The tanks had a 5 cm thick layer of fine sand or gravel and each male in each section was provided with a water pump system and aeration.

The experiments were performed from mid‐April to October, at random times. The reproductive behaviour of resident males was elicited by introducing one ripe female into the ‘arena’. Before trials, each female was left 12–24 h in the experimental tank for acclimatisation. Prior to recordings (approx. 15 min), electricity, water pumps and aeration were switched off to minimise ambient noise. To further reduce unwanted noise from the room light system, the ceiling light was switched off and the experimental tank was illuminated by LED light from the side of the aquarium. This procedure had no noticeable effects on fish behaviour. The male–female trials lasted approximately 30 min and began by removing one of the lateral partitions, allowing intersexual interaction. Note that the two lateral partitions were never removed at the same time, and the female always interacted with only one male from the same tank (i.e. two males from the same tank were used in separate trials). Between consecutive recording sessions 15–30 min pauses were included, with all the devices turned off. The eight territorial males were kept in the experimental tanks until October, after which they were returned to male community tanks. After each daily recording session, females were returned to their community tank (Figure S1). At the end of each recording session, males and females were measured for length and weight. Measurements were made using digital callipers CD‐15APX with a precision of 0.01 mm (Mitutoyo, Japan) and a digital scale (0.1 g precision). As a metric of male body condition, we calculated the condition factor Fulton's *K* [where *K* = (W/L_S_^3^) *10^5^] following Amorim et al. (2013).

### 2.2 Acoustic recordings and sound analysis

During acoustic recording sessions, a hydrophone (H2A‐XLR hydrophone, Aquarian Audio & Scientific; sensitivity: −180 dB re. 1 V μPa^−1^; frequency range ±4 dB from 0.01 to 100 kHz), was placed above the shelter (tunnel‐shaped plastic cover) and connected to a IRIG PRE preamplifier (Aquarian Audio & Scientific). Sounds were recorded using a ZOOM H4n portable digital audio recorder (16 bit/44.1 kHz sample rate; ZOOM). The hydrophone was placed within the attenuation distance from the emitter (less than 5 cm), and we obeyed the laboratory protocol for minimum resonant frequency for small glass tanks (e.g. 2.7 kHz for 170 L tanks, according to Akamatsu et al., 2002). Recordings were later band‐pass filtered (0.05–3 kHz) to improve S/N ratio and subsampled at 4 kHz, and further amplified (10 dB) for better auditory and visual inspection of the audio tracks. Digitalised sounds were analysed using Avisoft—SASLab Pro 5.2 Software (1024‐point FFT, FlatTop window; 100% frame; Avisoft Bioacoustics). Ten audio recordings (2.5 per male, each lasting approx. 30 min) were aurally and visually inspected. Not all sounds presented a good signal‐to‐noise ratio (S/N) for acoustic analysis. From 10 recordings presenting the best S/N ratio, we analysed 20 randomly selected sounds. Temporal features were measured from oscillograms, while frequency‐related variables were obtained from the logarithmic power spectra (FlatTop window, 512‐points FFT, 96.87% overlap; resolution 8 Hz). For sounds, we measured the following acoustic properties following Malavasi et al. (2008) and Amorim et al. (2013): (1) sound rate (SR, number of sounds emitted in 1 min from the start of sound production); (2) sound duration (DUR, total duration of the call, s); (3) number of pulses (NP); (4) pulse repetition rate (PRR; NP divided by DUR and multiplied by 1000, Hz); (5) pulse duration (PD; measured from the first to the last cycle in the pulse, ms); (6) pulse period (PP; average peak‐to‐peak interval of consecutive pulses, ms); (7) frequency modulation (FM, after the sound has been divided into three temporally identical sections, FMi—initial, FMm—middle and FMf—final, frequency modulation was calculated as the difference between the final and initial pulse repetition rate and expressed in Hz; FMi, pulse repetition rate of the initial section of a sound and FMf, pulse repetition rate of the final section of a sound); (8) peak frequency (PF, the peak with the highest energy from the logarithmic power spectrum function, Hz). In order to follow the previous recording protocols as closely as possible (Amorim et al., 2013; Amorim & Neves, 2007), we also calculated the vocal activity parameters per male: (i) sound rate (number of sounds produced per min), (ii) maximum sound rate (maximum number of sounds emitted in 1 min) and (iii) calling effort (percentage of time spent calling, i.e. sound production in seconds divided by the duration of the recording in seconds). Despite the fact that the variables PP and PRR indicate the pulse repetition pattern, they were deliberately indicated separately here to facilitate comparisons with the goby literature on sound production.

### 2.3 Video recordings and analysis of behavioural categories

During acoustic‐visual recordings, a second hydrophone (HTI‐96‐Min, High Tech Inc., sensitivity: −201 dB re. 1 V μPa^−1^, frequency response 2 Hz to 30 kHz), placed less than 3 cm from the nest opening, was connected directly to a video camcorder (Canon Legria FS200, 41x digital zoom, 25 frames/s) to directly synchronise acoustic and visual signals into a uniform dataset for subsequent analysis. The camcorder was mounted on a stand and positioned approx. 40 cm from the front of the experimental glass tanks. Courtship behaviour began when the females entered the male territory at a distance of <5 cm from the male's nest, while the pre‐spawning phase was observed when the ripe female entered the male's nest. The spawning phase began with the female turning upside‐down in the nest numerous times in short succession and started circling the ceiling. Male behaviours and the associated sound emissions were observed in four soniferous males during seven recording sessions and analysed using Solomon Coder (ver. beta 19.08.02). The ripe females were chosen for the recording sessions according to two indicators of their readiness for spawning (belly and eye colouration).

Behavioural categories expressed by the males were classified following the literature (Amorim et al., 2013; Amorim & Neves, 2007, 2008; Malavasi et al., 2009). We identified nine male behavioural categories within three distinct reproductive phases in *O. croaticus* (Table S1):

- Courtship phase, performed by the male outside the nest: Chase, Lead, Approach and Circling;
- Pre‐spawning phase, performed by the male within the nest: Nest display, Frontal display, Nest rubbing, Pre‐mating;
- Spawning phase, performed within the nest: Spawning.

Spawning was considered when the female repeatedly performed the upside‐down or belly‐up position, associated with oviposition. In some cases, Nest display and Frontal display were performed by the male occupying the nest with or without a female inside. However, Nest rubbing, Pre‐mating and Spawning were always performed by the male when the female was inside the nest. In Solomon Coder, two datasets were analysed separately and then compared. First, behaviour (frequency (*n* min^−1^) and duration (in s)) was scored in the video recordings with sound production. We noted the total number of sounds emitted per behavioural category for each soniferous male. Secondly, we analysed eight video recordings (two per male) containing the behaviours of the same four tested males, but when they did not produce sounds (i.e. males were silent for the entire recording period). By having these two datasets, we investigated the differences in frequencies of behavioural categories in males when they engaged in sound production and when they did not. In total, we used eight males in our experiments, but four were unresponsive (i.e. did not perform courtship behaviour or sounds), resulting in insufficient data for further analyses. We analysed videos for the following behavioural parameters: male behaviour rate (the total number of behavioural categories per min) (1) co‐occurring with sounds or (2) not co‐occurring with sounds; number of times a female entered the male's nest (3) accompanied with sounds or (4) without sounds; (5) total behaviours (number of behavioural categories per video recording).

### 2.4 Anatomical analysis

For anatomical dissections, additional *O*. *croaticus* individuals were collected in October 2020 from the same watercourse near the village Brečići (43°7′11.30″ N, 17°29′4.03″ E) using electrofishing. Five individuals were collected, of which three males (40–50 mm *L*_T_) were immediately euthanised with an overdose of MS‐222 (tricaine methane sulphonate; Pharmaq), and stored for 1 week in 7% formaldehyde fixative solution and then transferred to 70% ethanol. Specimens were dissected and examined with a Wild M10 binocular microscope (Leica Camera, Leica) equipped with a camera lucida to study the anatomy of the putative sound‐producing mechanism. Since earlier research on gobies highlighted the role of the pectoral girdle and (pectoral) fins in sound production, dissections primarily addressed the muscles related to this body part. The nomenclature used to designate muscular parts was based on earlier research (Adriaens et al., 1993; Parmentier et al., 2013, 2017; Winterbottom, 1974). Additionally, one specimen was subjected to micro‐computed tomography (μCT) scanning to visualise the fish skeleton at the level of the neurocranium and pectoral girdle. Scanning was completed using a RX EasyTom (RX Solutions; http://www.rxsolutions.fr), with an aluminium filter. Images were generated at 75 kV and 133 μA, with a frame rate of 12.5, 5 average frames per image. This procedure generated 2897 images at a voxel size of 10 μm. Reconstruction was performed using X‐Act software from RX Solutions. Segmentation, visualisation and analysis were performed using Dragonfly software (Object Research Systems (ORS) Inc, 2019; software available at http://www.theobjects.com/dragonfly). Three‐dimensional (3D) 16‐bit images were produced and subsequently converted into 8‐bit voxels using ImageJ (Abramoff et al., 2004). Three‐dimensional processing and rendering were obtained after semi‐automatic segmentation of the body using a ‘generated surface’, according to the protocols described by Zanette et al. (2013). Direct volume renderings (iso‐surface reconstructions) were used to visualise a subset of selected voxels of the anterior skeleton in AMIRA 2019.2.

### 2.5 Acoustic comparison among soniferous sand gobies

The sounds of seven soniferous sand gobies, *Knipowitschia panizzae* Verga, 1841, *Ninnigobius canestrinii* (Ninni 1883), *Orsinigobius punctatissimus* (Canestrini 1864), *Pomatoschistus marmoratus* (Risso 1810), *P. pictus* (Malm, 1865), *P. microps* (Krøyer, 1838) and *P. minutus* (Pallas 1770), were recorded and characterised by previous studies (Amorim et al., 2013, 2018; Bolgan et al., 2013; Lugli et al., 1995; Lugli & Torricelli, 1999; Malavasi et al., 2008; Pedroso et al., 2013). However, these acoustic data were never combined into a single phylogenetic dataset and analysed interspecifically. Therefore, we studied interspecific acoustic variability of soniferous sand gobies (*P. marmoratus* was separated geographically into two populations, Italian and Portuguese). Briefly, the species were caught in the past by authors of previous studies either from brackish habitats in north Adriatic Sea (*K. panizzae*, *P. marmoratus* and *N. canestrinii*), from freshwaters of north‐west part of Reggio Emilia Romagna, Italy (*O. punctatissimus*; Lindström & Lugli, 2000; Lugli et al., 1995, 1997; Lugli & Torricelli, 1999), from Portuguese marine/brackish waters (Amorim et al., 2013, 2018; Bolgan et al., 2013) or the west coast of Sweden (Pedroso et al., 2013). Sound recordings gathered from the previously conducted laboratory experiments were re‐analysed to allow for interspecific comparison with a minimal measurement experimental error. All investigated sand gobies produced pulsatile sounds, thus enabling acoustic interspecific comparisons. The dataset was composed of 36 individuals of eight soniferous sand gobies including *O. croaticus* (min–max: 3–5 individuals, except for a single individual of *P. microps*), with at least three sounds recorded per individual. In total we calculated the means for five acoustic variables (temporal: DUR in ms, NP, PRR in Hz; spectral: PF and FM, both in Hz) for each individual. Since gobies included in the current study were recorded at different water temperatures (range: 15.8–22.6°C) and it is well known that the ambient water temperature affects fish acoustic signals (Ladich, 2018; Vicente et al., 2015), we conducted two separate multivariate analyses: the first involving the complete dataset (all five acoustic features for each species), and the second excluding the temporal features (DUR and PRR) known to be influenced by water temperature (Lugli et al., 1996; Vicente et al., 2015).

### 2.6 Statistical analysis

The statistical analyses were performed by combining the sounds from multiple individuals into a single dataset. Outliers and extremes were detected visually from the boxplot and were eliminated from the dataset if necessary. Since the data were not normally distributed for some variables from the raw intraspecific dataset (Shapiro‐Wilk test, *p* < .05), we used non‐parametric tests. For pairwise comparisons between soniferous *O. croaticus* males, we employed the Kruskal‐Wallis rank sum test *H* followed by pairwise Dunn's multiple comparison test with Bonferroni correction for the *p*‐values. Additionally, the *Chi*‐square (χ^2^) was used to test for independence of behaviour from sound production. In this test, the residuals from the χ^2^ were used to determine which behaviours were positively related to sound production. Kruskal‐Wallis *H* test was used to compare the mean behavioural variables (calling rate, behaviour rate, n. of female nest entrances) between soniferous males. Wilcoxon signed‐rank test was performed to compare the two dependent samples, that is, mean behavioural variables (behaviour rate and female nest entrance) of males when they produced sounds and when they were silent. Additionally, Wilcoxon test was used to compare the frequency and duration of courtship and pre‐spawning phases between males.

For the interspecific comparisons, the means of individual acoustic properties of soniferous sand gobies were compared with the Kruskal‐Wallis *H*‐test, since the data were not normally distributed (Shapiro‐Wilk test, *p* < .05). To quantify interspecific acoustic variability among the soniferous sand gobies from our study, we used a multivariate approach. PCA, based on the correlation matrix, was performed on transformed and standardised individual means of five sound variables (temporal: DUR, NP, PRR; spectral: PF and FM) to assess overall acoustic variability between sand gobies, and additionally to recognise acoustic variables explaining the observed variance. To assess the percentage of successful classification of the sounds assigned to the correct goby species, and to maximise the separability among taxa, we used linear discriminant analysis (LDA). Two different LDAs were performed, first with the complete dataset (five acoustic variables for each species) and then removing the temperature‐dependent features (DUR and PRR). Due to the FM's negative raw values, we added a positive factor to this feature so that we could use it in the comparative analyses. Our results were presented as means (*x̄*) ± standard deviation (SD), while the level of significance for inter‐ and intraspecific comparisons was 5% (*α* = 0.05). Statistical analyses were performed in STATISTICA® (v. 13.6.0., TIBCO, USA), Past (v. 4.11) and R Studio (2022.07.0) software.

### 2.7 Permits

*Orsinigobius croaticus* is legally protected by law as an endangered taxon in Croatia (Official Gazette of the Republic of Croatia, 2016). In addition, it is an endemic species with very limited distribution. As a result, the number of individuals employed in the laboratory experiments was kept to a minimum (less than 15) to prevent possible effects on the natural population of this species. The sampling by electrofishing for scientific purposes in the natural habitat was approved by the Ministry of Agriculture (permit number 525‐13/0545‐19‐2), while all laboratory experimental protocols were approved by the Bioethics and Animal Welfare Committee of the Faculty of Science, University of Zagreb (permit number 251‐58‐10617‐21‐147).

## 3 RESULTS

### 3.1 Sound production and intraspecific sound signal structure

**FIGURE 1:**
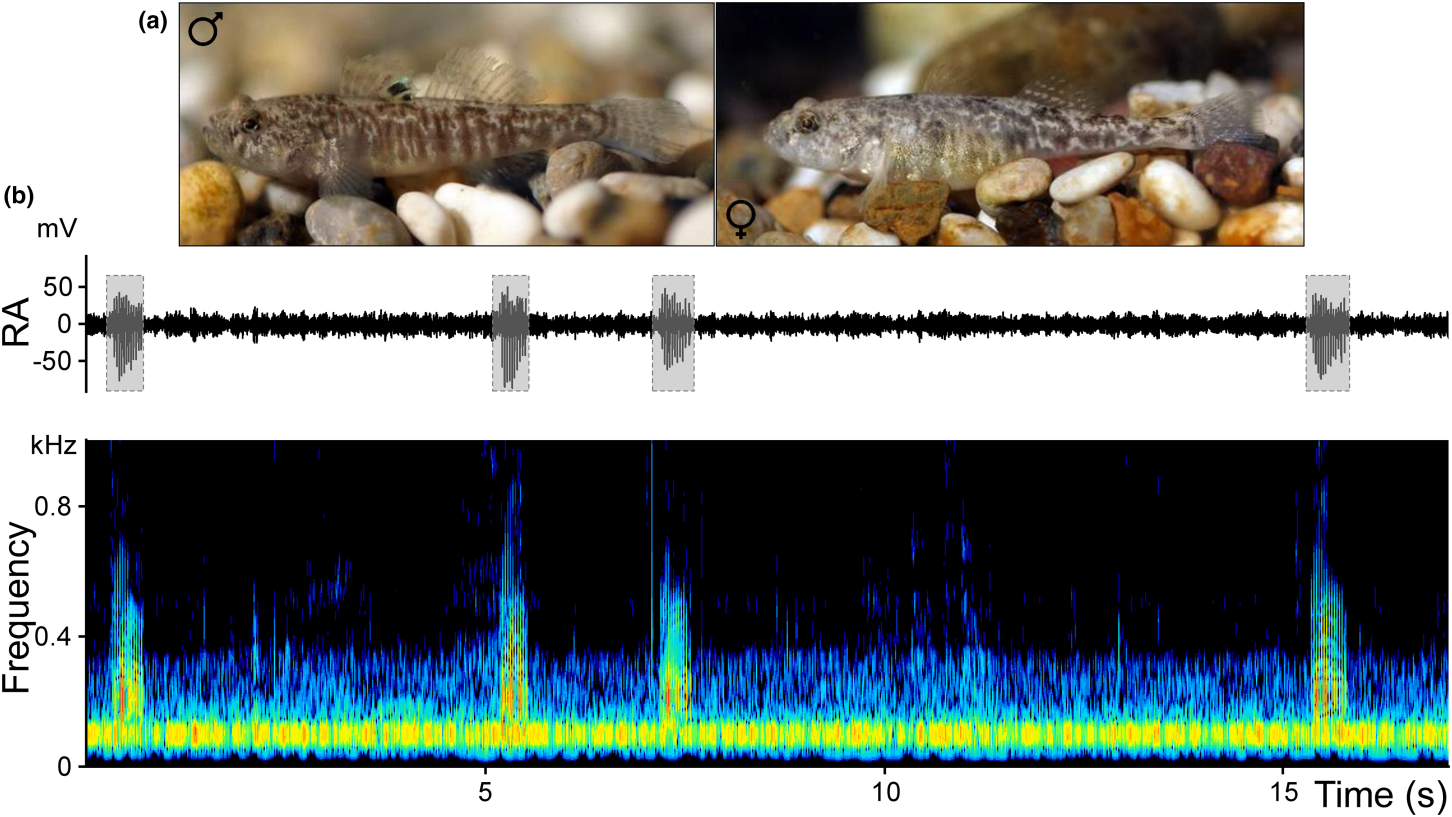
(a) Male and female of Neretva dwarf goby, *Orsinigobius croaticus*. Photos of individuals from Horvatić et al. (2017), (b) Sound production of *O. croaticus* males. The 17‐second recording clip depicts the oscillogram (top) and spectrogram (bottom) of four pulsatile sounds produced by a male goby (grey dashed area). In the spectrogram, warmer colours indicate higher acoustic energy (orange is highest and blue is lowest). The horizontal band at low frequency (approx. 100 Hz) corresponds to ambient noise stemming from the ambient laboratory conditions. Spectrogram parameters: FlatTop window, 1024 length FFT; 25% frame size; 93.75% overlap; resolution: 4 Hz. RA, relative amplitude.

**FIGURE 2:**
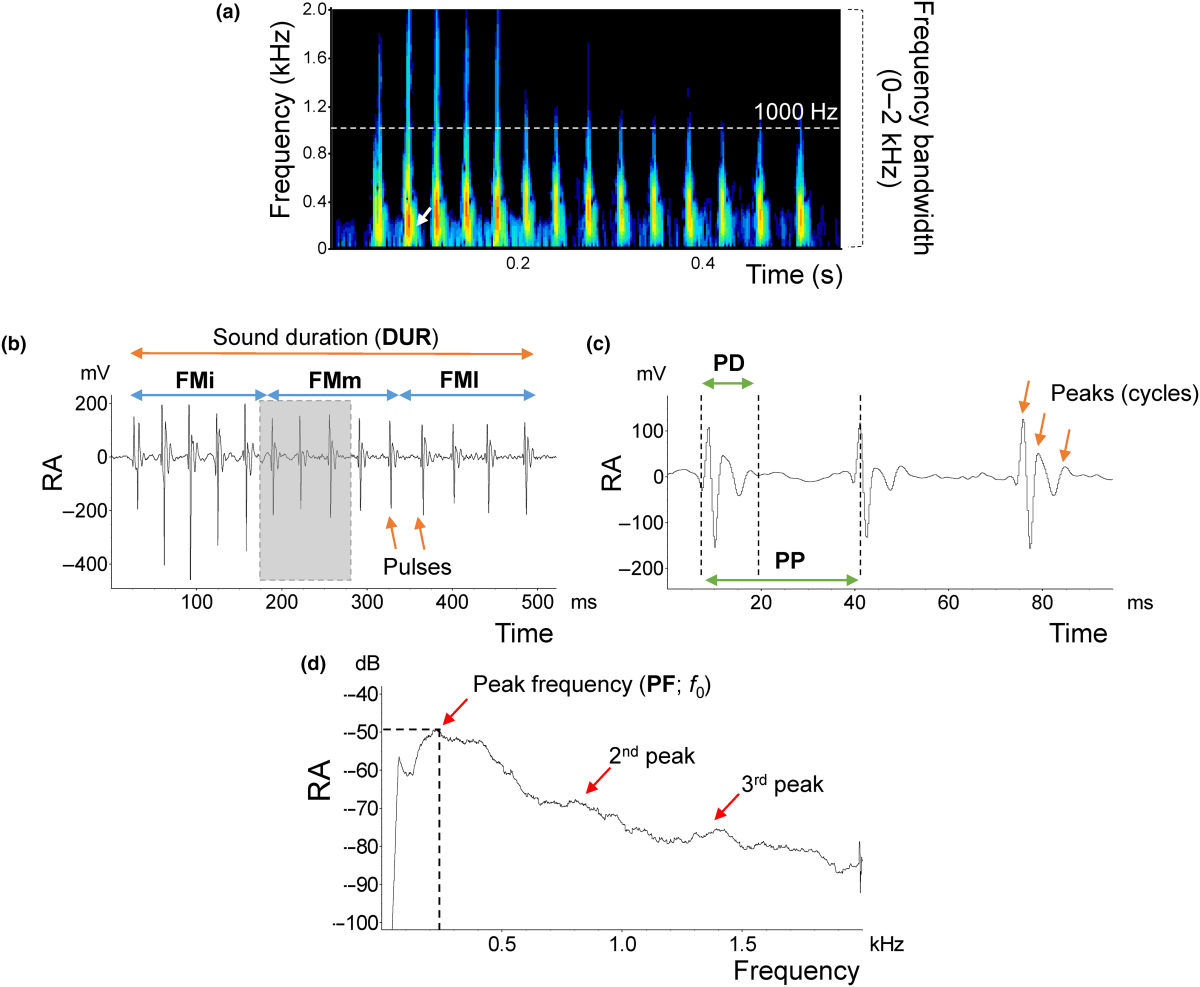
Structure of a pulsatile sound produced by *Orsinigobius croaticus*. Diagram illustrating the acoustic variables measured from the pulsatile sounds. (a) Spectrogram and (b) oscillogram of the pulsatile sound; (c) oscillogram of isolated pulses (6–8) in grey; (d) power spectrum of the pulsatile sound from (a). DUR, sound duration (total length of the call, measured in milliseconds); NP, number of pulses; PRR, pulse repetition rate (NP divided by DUR and multiplied by 1000 Hz); PD, pulse duration (ms); PP, pulse period (average peak‐to‐peak interval of consecutive pulses, ms); FM, frequency modulation (after the sound has been divided into two sections, FMi and FMf, frequency modulation was calculated as the difference between the final and initial pulse repetition rate and expressed in Hz); FMi, frequency modulation—initial (pulse repetition rate of the initial section of a drum); FMm, frequency modulation‐middle, FMf, frequency modulation—final (pulse repetition rate of the final section of a drum); PF, peak frequency (obtained as a peak with the highest energy from the logarithmic power spectrum function, Hz; white arrow in spectrogram), *f*_0_ also correspond to the fundamental frequency. Spectrogram parameters: FlatTop window, 256 length FFT; 25% frame size; 93.75% overlap; resolution: 4 Hz. RA, relative amplitude.

Males of *O. croaticus* produced a single type of acoustic signal, pulsatile sound, during intersexual (male–female) interactions conducted within the reproductive season (April–October). Four resident males ($\bar{x}$ ± SD = 49.1 ± 0.8; range: 48.0–50.0 mm *L*_T_; 40.9 ± 1.8; range: 38.9–42.2 mm *L*_S_; 1.2 ± 0.1; range: 1.1–1.4 g *W*; 1.7 ± 0.1; range: 1.5–1.9 Fulton's *K*) produced sounds when interacting with females, while the other four males remained silent and did not court. We recorded 372 sounds produced by the four males (93 sounds per male). Sounds were produced in an irregular pattern (7.7 ± 1.4; range: 6–10 sound min^−1^; Figure 1; Table 1). The sounds are short‐duration signals, lasting 450 ms (442.0 ± 132.6 ms), and composed of a variable number of short pulses (14.2 ± 4.0) of around 15 ms (14.5 ± 1.9 ms; Figure 2a–d; Table 1). Pulse structure differed between sounds, exhibiting one to three peaks with variable amplitude. Generally, the amplitude of a sound changed gradually, first increasing and then steadily decreasing throughout the sound, with the first two or three pulses being the highest in amplitude (Figure 2a–d). The pulse repetition rate varied from 26.0 to 38.0 Hz (32.5 ± 1.6 Hz), while the pulse period (PP) averaged 32 ms (31.9 ± 1.4; range ms). PP changed with water temperature, with higher values occurring at lower temperatures. In the pulsatile sounds, last PP was always longer than the remaining PPs. Peak frequency varied from 89 to 340 Hz (137.4 ± 38.3 Hz), although several higher frequency components were also present, especially in the range 0.5–1.5 kHz. Energy extended from 0.05 to 2 kHz (Figure 2a,d; Table 1), with most of the sound energy concentrated within 0.05–0.6 kHz. Frequency modulation of the sounds ranged from 0.7 to 1.1 Hz. Additionally, the calling effort varied between males from 0.37 to 0.60 of sound production/s of recording (0.49 ± 0.09), indicating that some individuals emitted sounds more frequently than others (Table 1). Interestingly, sounds were never organised in bursts, which are usually composed of several consecutive sounds produced with regular inter‐sound intervals, as observed in some sand gobies. During our intersexual acoustic experiments, no females produced a sound.

**TABLE 1:** Descriptive statistics of sound acoustic parameters produced by male *Orsinigobius croaticus*.

Acoustic parameters	$\bar{x}$	SD	Range	*H*	*p*‐value
DUR (ms)	442.1	158.7	156.8–952.9	22.9	**<.05**
NP	14.3	5.1	5.0–32.0	24.5	**<.05**
PD (ms)	14.6	2.1	9.8–23.0	8.1	**<.05**
PRR (Hz)	32.5	2.6	26.0–38.1	36.9	**<.05**
PP (ms)	32.0	2.8	27.7–37.6	23.7	**<.05**
PF (Hz)	137.4	55.5	89.0–340.8	34.7	**<.05**
FMi	39.0	4.1	31.4–60.0	38.1	**<.05**
FMf	34.1	3.7	26.1–45.4	29.9	**<.05**
FM (Hz)	0.9	0.1	0.7–1.1	12.8	**<.05**
SR (no. of sounds/min)	4.7	2.1	1.3–7.8	8.6	**<.05**
Calling effort	0.5	0.1	0.3–0.6	5.7	>.05

*Note*: For each parameter, mean ($\bar{x}$), standard deviation (*SD*) and range were reported, with the corresponding results from the intraspecific Kruskal‐Wallis rank sum test and *p*‐value.

Descriptive statistics are based on 20 sounds per male presenting the best S/N ratio (*N* = 4, *n* = 80). *H*‐values are the results of Kruskal–Wallis tests comparing sound parameters among males. Bolded *p*‐values indicate the feature that differed between males according to the significance level of .05. For the abbreviations of acoustic properties, see Section 2.

Intraspecifically, soniferous *O. croaticus* males differed in all acoustic features (Kruskal‐Wallis *H*‐test, SR χ^2^ = 8.59, DUR χ^2^ = 22.87, NP χ^2^ = 24.53, PRR χ^2^ = 8.5936.87, PD χ^2^ = 8.07, PP χ^2^ = 23.73, PF χ^2^ = 34.68, FMi χ^2^ = 38.05, FMf χ^2^ = 29.92, FM χ^2^ = 12.81; df = 3; *N* = 80; *p* < .05 for all features) except for calling effort (Kruskal‐Wallis *H*‐test, χ^2^ = 5.67; df = 3; *N* = 7; *p* > .05; Table 1).

### 3.2 Reproductive ethology and association with sounds

**FIGURE 3:**
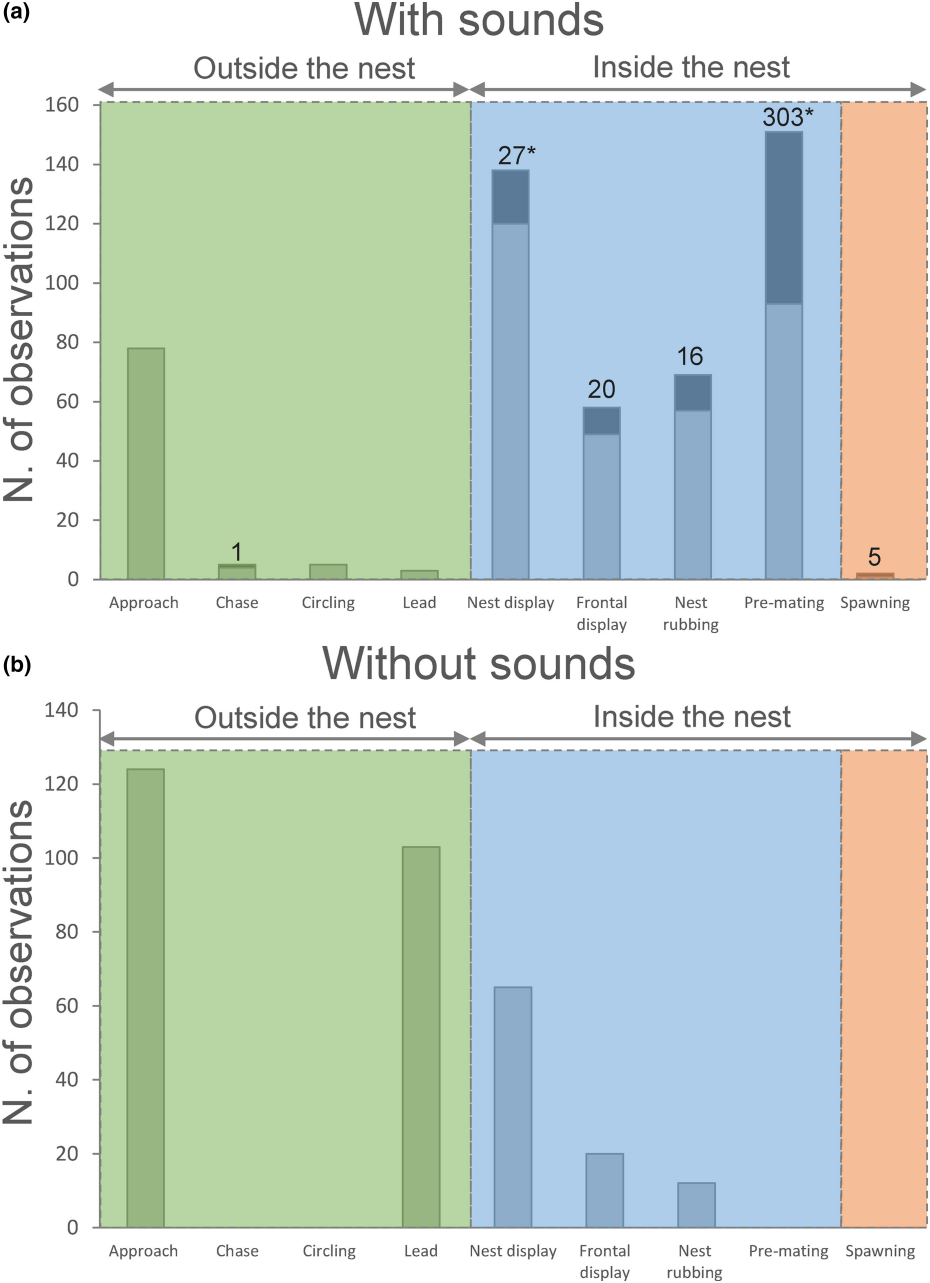
Acoustic behaviour of *Orsinigobius croaticus* males during sexual interactions expressed through nine behavioural categories. In (a), sound production was observed in certain categories (Chase, Nest display, Frontal display, Nest rubbing, Pre‐mating and Spawning), which were accompanied by sound emission (i.e. the dark grey bar at the top of the column). In (b), no sound production was observed. In (a) and (b), the light grey colour of the column indicates the number of documented courtship behavioural acts. Asterisk (*) indicates the significant association of behavioural categories with sound production. Numbers above columns indicate the total number of recorded sounds per each behavioural category. The green area encompasses the courtship phase performed outside of the nest, the dashed blue area highlights the pre‐spawning phase while the dashed orange area indicates the spawning phase of reproduction, displayed in the nest.

The frequency, duration and overall percentage of male behavioural categories were scored for four soniferous males in different sessions: with sound production and silent. The first dataset included seven video recordings (210 min) where at least one sound occurred per recording by each male. Overall, we observed 410 behavioural categories (102.5 per male). The behavioural categories Nest display (29.3%), Pre‐mating (22.7%) and Approach (19.1%) were most frequently observed while Circling (1.2%), Chase (1.0%) and Lead (0.7%) were rarely recorded (Figure 3). Of the 410 behavioural categories (from four males), 99 categories (24.1%) were accompanied by sound production. Sound production was documented during trials for the pre‐spawning phase: Pre‐mating (303 sounds), Nest display (27 sounds), Frontal display (20 sounds), Nest rubbing (16 sounds) and for the spawning phase: Spawning (five sounds). Sounds did not co‐occur with the courtship phases: Approach, Circling and Lead (Figure 3). During four Chase, only one sound was recorded. Only one spawning act (Spawning) was observed in this study, during which five sounds were produced. Unfortunately, spawning sounds were not used in the comparative purposes due to their limited occurrence. The chi‐square (χ^2^) test of independence indicated that behavioural categories Nest display and Pre‐mating were significantly associated with sound production (χ^2^ = 138.3; df = 5; *N* = 99; *p* < .05; residual score: 1.5 and 41.5, respectively), while other categories failed to support this hypothesis (Figure 3).

To compare male behaviour when soniferous or silent, a second dataset of eight video recordings was considered (190.5 min) of the same four males but in which no sound production was documented. In these recordings, we observed 324 male behavioural categories (averaging 81.0 per male), of which Approach (38.3%), Lead (31.8%) and Nest display (20.1%) were the most frequent categories (Figure 3). Contrary, Chase, Circling, Pre‐mating or Spawning were not documented within these recording sessions. In general, there is an obvious dissimilarity between the frequency of the behavioural categories that were or were not accompanied by sounds. Specifically, Pre‐mating, one of the two behaviours significantly associated with sound production, decreased from an average of 22.7% in the trials with sounds to 0% in the trials without sound. In addition, Nest display, Nest rubbing and Frontal display categories produced during sound emission decreased in frequency in experiments without the sounds (Nest display: from 29.3% to 20.1%; Nest rubbing: from 13.9% to 3.7%; Frontal display: from 12% to 6.2%). On the other hand, Approach and Lead were more frequent during the silent sessions (31% and 38%, respectively) than during sound production (19% and 0.7%, respectively; Figure 3). Overall, the behavioural rate decreased from 55.8% to 44.1% when males produced sounds in comparison to when they were silent (soniferous vs. silent males: means 2.79 vs. 1.55), though the differences were not significant (*Wilcoxon* signed‐rank test, *N* = 14; *p* > .05). Importantly, the number of times the females entered the male nest differed significantly between the two datasets (3.71 vs. 0.71), as female nest entrance was more frequent when males produced sound than when they were silent (*Wilcoxon* signed‐rank test, *N* = 16, *p* < .05). Finally, the two males receiving the most female entries were the largest in size (41.7 and 42.2 mm SL). These two males produced the sounds with highest values of NP (>13 pulses), FMi (>38.5 Hz) and PRR (>33.5 Hz).

Considering the sessions in which sound occurred, the frequency of occurrence of behaviours between the courtship (Chase, Lead, Approach and Circling) and pre‐spawning (Nest display, Frontal display, Nest rubbing, Pre‐mating) phases of reproduction did not differ (*Wilcoxon* signed‐rank test, *N* = 8; *p* > .05), though their duration did (*Wilcoxon* signed‐rank test, *N* = 8; *p* < .05). Generally, males exhibited courtship‐related behaviours less frequently and for a shorter period compared to pre‐spawning behaviours.

### 3.3 Anatomical findings and movements during sound production

**FIGURE 4:**
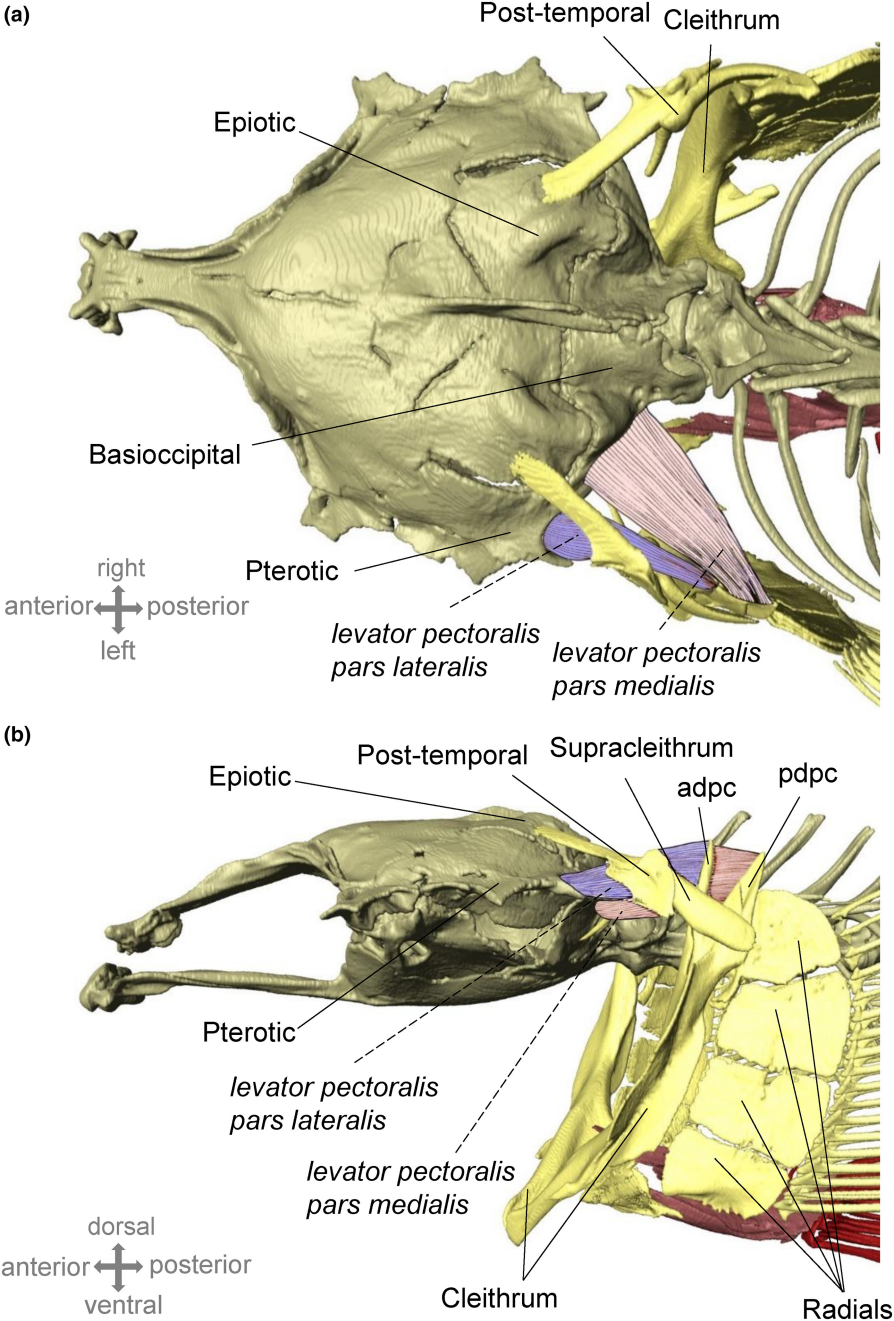
Micro‐computed tomography (μCT) scan of the osseous structures and sonic muscles of the putative sound‐producing mechanism in *Orsinigobius croaticus*. (a) Dorsal view of the neurocranium, pectoral girdle and sonic muscles (right side), (b) Left lateral view of the neurocranium and pectoral girdle, with sound‐producing *levator pectoralis* muscles indicated in red (*pars medialis*) and purple (*pars lateralis*). adpc, anterior dorsal process of the cleithrum, pdpc, posterior dorsal process of the cleithrum.

The pectoral girdle of *O. croaticus* was subjected to μCT scanning and dissection to identify the various osseous structures and muscles that may be involved in sound generation. From μCT scans, three functional units were distinguished in the skeletal part of the pectoral girdle of *O. croaticus*: the shoulder girdle (composed of the post‐temporal, the supracleithrum and the cleithrum bones) dorsally attached to the neurocranium, the shoulder plate (i.e. four large radials) and the fin plate, made up of fin rays articulated with the shoulder plate (Figure 4). On the dorsal tip of the cleithrum, anterior and posterior processes are present. The supracleithrum articulates with the post‐temporal and the cleithrum, connecting with the cleithrum bone at its dorsal tip. The post‐temporal is made up of a basal plate and two rostrally oriented processes (a ‘fork’) with dorsal and lateral attachments to the neurocranium. The rostral tip of the dorsal process is flattened and firmly attached to the epiotic bone. Putative sound‐producing muscles were observed during the dissection and were found originating on the neurocranium and inserting on the pectoral girdle (Figure 4). The *levator pectoralis* muscle is divided into two bundles: the *pars lateralis* and the *pars medialis*. The *pars lateralis* originates on the posterior part of the pterotic and inserts on the anterior dorsal process of the cleithrum. The *pars medialis* is the thicker of the two muscles. It originates on the posterior part of the basioccipital and inserts on the medial part of the posterior dorsal process of the cleithrum.

Moreover, video recordings allowed to highlight characteristic fish movements during sound production, especially concerning the head region and fins. During sound production, soniferous males would usually stop swimming and suspend the body on the fused pelvic fins. In addition, the pectoral fins were abducted, and the rays spread during sound emission. The male performed a lateral body quiver starting from the head to the tail (including dorsal fins), while the dorsal fins (both first and second) were erected prior to the production of the first pulse. Then the male would rapidly elevate the head and perform lateral head motions (while spreading the buccal and opercular cavities), accompanied by sound emission. The mouth was closed during the period of emission, though the anterior part of the branchial basket was slightly uplifted. Rarely, sound emission occurred during the head elevation phase.

### 3.4 Interspecific acoustic diversity in soniferous sand gobies

**FIGURE 5:**
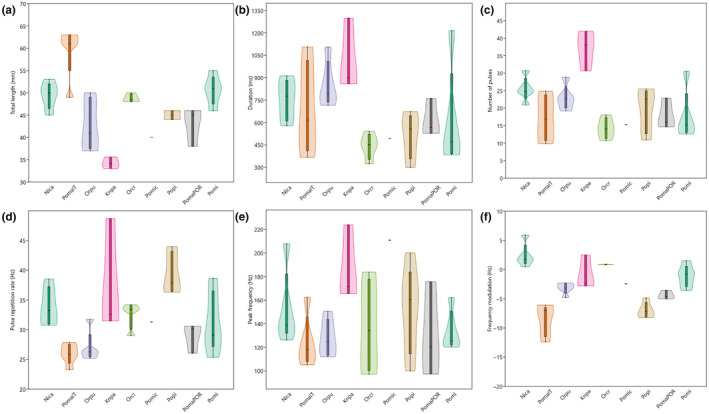
Violin plot with box plot of five acoustic variables and size (total length, in mm) measured from eight species of soniferous sand gobies from this study (*Pomatoschistus marmoratus* was divided into two geographically separated populations). Each colour represents a different species. The violin plot shows the kernel density plot (i.e. continuous histogram) for each variable. For each sample, the 25%–75% quartiles are drawn using a box. The median is shown with a horizontal line inside the box. The minimal and maximal values are shown with short horizontal lines (‘whiskers’). Species codes: Nica ‐ *Ninnigobius canestrinii*; PomaIT ‐ *Pomatoschistus marmoratus* (Italian population); Orpu ‐ *Orsinigobius punctatissiumus*, Knpa ‐ *Knipowitschia panizzae*; Orcr ‐ *Orsinigobius croaticus*; Pomic ‐ *Pomatoschistus microps*; Popi ‐ *Pomatoschistus pictus*; PomaPOR ‐ *Pomatoschistus marmoratus* (Portuguese population); Pomi ‐ *Pomatoschistus minutus*.

Eight soniferous sand gobies used in our analysis, produce pulsatile sounds, thus enabling acoustic interspecific comparisons (Table S2). Interspecific pairwise comparisons revealed interspecific differences in the acoustic features DUR, NP, PRR and FM (Kruskal‐Wallis *H*‐test, χ^2^ = 15.97–30.19; df = 8; *N* = 36; *p* < .05), while they did not differ in PF (Kruskal‐Wallis *H*‐test, χ^2^ = 11.54; df = 8; *N* = 36; *p* > .05; Figure 5a–f). On average, *P. marmoratus* (Portuguese), *O. punctatissimus*, *P. microps* and *K. panizzae* were the smallest in size (34–43 mm *L*_T_), while *P. minutus*, *N. canestrinii* and *P. marmoratus* (Italian) were the largest species (50–59 mm *L*_T_). In most cases, *K. panizzae* differed significantly from other species, especially in DUR and NP (Dunn's multiple comparison test, *p* < .05). Regarding PF, *P. microps* had the highest mean values, alongside *K. panizzae* (Dunn's multiple comparison test, *p* < .05). Finally, *P. marmoratus* (Italian population) and *P. pictus* differed significantly from the rest of the species having lower values of FM, while other species presented upward‐ or downward‐modulated sounds (Dunn's multiple comparison test, *p* < .05; Figure 5a–f).

**FIGURE 6:**
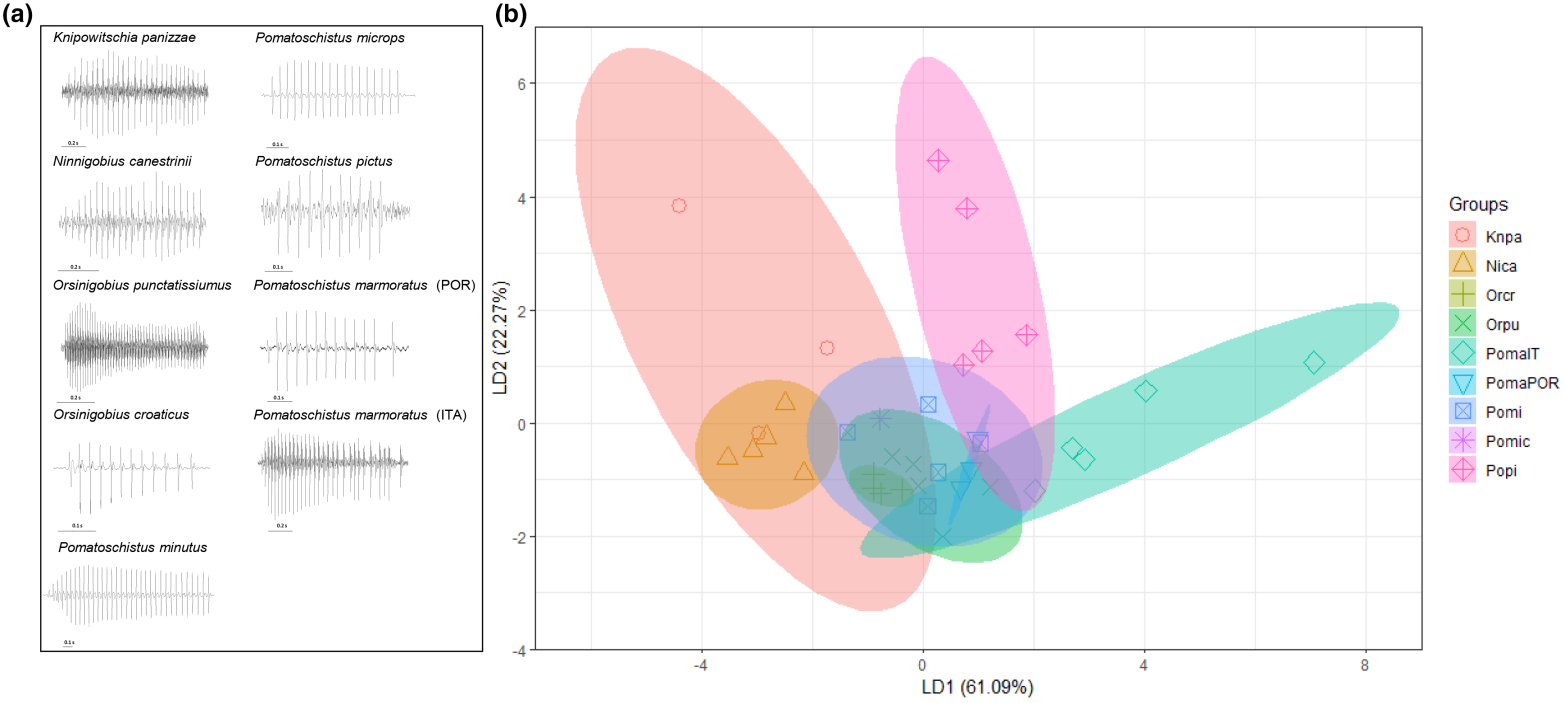
(a) Representative waveforms of pulsatile sounds produced by the soniferous sand gobies. Species codes: Nica ‐ *Ninnigobius canestrinii*; PomaIT ‐ *Pomatoschistus marmoratus* (Italian population); Orpu ‐ *Orsinigobius punctatissiumus*; Knpa ‐ *Knipowitschia panizzae*; Orcr ‐ *Orsinigobius croaticus*; Pomic ‐ *Pomatoschistus microps*; Popi ‐ *Pomatoschistus pictus*; PomaPOR ‐ *Pomatoschistus marmoratus* (Portuguese population); Pomi ‐ *Pomatoschistus minutus*. Horizontal scale bars represent time interval (in seconds). (b) Bi‐plot of LD1 and LD2 from the linear discriminant analysis, using five standardised and transformed acoustic variables (DUR, NP, PRR, PF and FM) for eight sand goby species. On the *X* axis, LD1 explains 61.09% of the trace proportion (i.e. percentage of separation), while on the *Y* axis, LD2 explains 22.27% of the trace proportion.

In PCA, the first two principal components of the PCA explained cumulatively 69.79% of the variation, with PC1 and PC2 explaining 39.28% and 30.51% of the variation, respectively. On the PC1 and PC2 scatterplots, although several species are clearly separated based on the acoustic features of their sounds, most of the plots overlap (Figure S2). PC1 was strongly associated with DUR (−0.69) and NP (−0.67), while PRR (−0.65) and PF (0.58) mostly contributed to PC2. We performed two LDA analyses, first with the complete dataset (five acoustic variables DUR, NP, PRR, PF and FM) and the second excluding the temperature‐dependent features (DUR and PRR), to test for sound classification into correct groups (i.e. species). In the first LDA, the first two axes accounted for a discrimination of 83.36%, with LD1 accounting for 61.09% and LD2 for 22.27%. LDA successfully attributed the most sounds of a sand goby to the correct species according to five acoustic parameters, with a correct interspecific classification rate of 86.11%. For some goby species, a contingency table supports the 100% level of correct classification of sounds (*N. canestrinii*, *O. croaticus*, *O. punctatissimus*, *P. pictus* and *P. microps*), while for the remaining species lower levels were achieved (67% for *K. panizzae*, 80% for Italian and 67% for Portuguese *P. marmoratus*, 60% for *P. minutus*). In the LDA bi‐plot, species clusters overlap, but not significantly, with some taxa occupying relatively isolated positions along the LD axes (Figure 6). LD1 was significantly loaded with FM (0.32), while LD2 with PRR (−0.47) and PF (−0.28). To exclude the effect of water temperature on the interspecific acoustic classification success by LDA, we carried out a second LDA, including only the three acoustic features that are known to be unaffected by water temperature, namely NP, PF and FM. In this second LDA, axis 1 and 2 accounted for 95.49% of discrimination, with LD1 axis accounting for 66.88% and LD2 for 28.61%. However, the second LDA was less successful than the first LDA in accurately classifying the sounds of sand gobies, with a 69.44% rate of correct interspecific classification. Again, some species (*K. panizzae*, *P. microps* and *N. canestrinii*) achieved 100% classification, while the remaining species were misidentified in different percentages in comparison to the first LDA (75% for *O. croaticus* and 80% *O. punctatissimus*, 66% for *K. panizzae*, 40% for Italian and 33% for Portuguese *P. marmoratus*, 40% *P. minutus* and 80% for *P. pictus*).

## 4 DISCUSSION

### 4.1 Acoustic structure and sound characteristics

This study investigated for the first time the sound production and reproductive intersexual behaviour of a freshwater endemic Mediterranean goby, *Orsinigobius croaticus*, under laboratory conditions. Males of *O. croaticus* produced pulsatile sounds when interacting with females, during courtship, pre‐spawning and spawning phases of the reproductive behaviour. Males did not produce sounds in all trials and calling rate varied between males and with female proximity. When males were in close contact with females or the prospective female approached/entered the nest, the calling rate would significantly increase from a few up to 10 sounds per min.

The pulsatile sounds in sand gobies are composed of a variable number (range 5–32) of pulses (organised in pulse trains), which are considered the fundamental units of this acoustic signal (Lindström & Lugli, 2000; Zeyl et al., 2016). *Orsinigobius croaticus* acoustic signals are short and low‐frequency sounds (<500 ms, ~140 Hz) composed from a short number of sound pulses with an average duration and period of around 15 and 32 ms, respectively. Pulsatile sounds from *O. croaticus* were never organised in bursts, a state that is observed in other soniferous species such as *P. marmoratus*, *P. pictus*, *P. microps* and *P. minutus* (Amorim & Neves, 2007; Blom et al., 2016; Lugli & Torricelli, 1999).

In addition, pulsatile sounds of *O. croaticus* males differed in all acoustic features (except calling effort), and these acoustic differences among soniferous males might suggest the intraspecific acoustic variability of their reproductive sounds. Despite the small sample size, PD and PP differed significantly among males. In pulsed acoustic signals, PD can be related to body size and condition (Amorim et al., 2010) or temperature (Bennett, 1985; Vicente et al., 2015), while PP is often dependent on temperature, but also reflects phylogenetic affinities in fish groups such as pomacentrids, cichlids and sand gobies (Amorim et al., 2008, 2013; Myrberg et al., 1978; Vicente et al., 2015).

During intersexual acoustic experiments, females did not produce any sound or display any visible movements, such as upward head thrust or dorsolateral motion of the opercula, which would indicate possible sound production. Although sound emission in females was previously documented in some gobies, here we did not investigate intrasexual (female–female) interaction, which is known to trigger sound production (Horvatić et al., 2016; Ladich & Kratochvil, 1989).

Here we only detected one sound type‐pulsatile sounds. In agonistic or reproductive circumstances, some sand gobies have the capacity to emit not only one but few sound types (pulsatile and thumps, de Jong et al., 2016; Zeyl et al., 2016). There is still significant debate about why fish use various sound types during these encounters, and some speculate that each sound type may have a particular purpose (Amorim, 2006).

Finally, previous field or laboratory studies indicated that some of the acoustically active fish are nocturnal, dusk or dawn callers (Bertucci et al., 2021; Chang et al., 2022; Jublier et al., 2019). There is a lack of knowledge about the general daily activities and behaviour of *O. croaticus*. Since we only conducted the acoustic experiments during the daytime (10 AM–19 PM), it is probable that the time of the recordings could had an impact on the quantity of acoustic signals that were emitted or the overall calling pattern. Repeating the additional recording sessions during dawn/dusk or night (7 PM–11 AM) might be interesting if *O. croaticus* is discovered to be a crepuscular or nocturnal species.

### 4.2 Sound production in relation to reproductive behaviour

In this study, *O. croaticus* males exhibited nine (visual) behavioural categories, confined to three distinct reproductive phases. The sound production in males was mostly associated with pre‐spawning behaviours in the nest. In addition, males exhibited courtship‐related behaviours less frequently and for a shorter period than pre‐spawning behaviours. These findings suggest that the sound production is important in the mating process in *O. croaticus*. Regarding the multimodal communication, soniferous *O. croaticus* males differed in the frequency and occurrence of displayed behavioural categories when producing sounds and when they were silent since most of the categories in the silent experiments were related to the courtship phase (outside the nest). Some behavioural categories, such as Pre‐mating, Chase, Circling and Spawning, were completely absent from silent experiments. When producing sounds, Pre‐mating and Nest display were the most frequent categories, indicating that males modulate their behaviour according to mate attraction investment. These findings could indicate that the multimodal signals, as produced by *O. croaticus* males, could convey a wider set of information to the prospective breeding females, rather than using only one signal type. Indeed, males of different species, such as *P. pictus*, make a suite of signals from one or more modalities that females may use in mating decisions (Amorim et al., 2013; Amorim & Neves, 2007; Bro‐Jørgensen, 2010). Multimodal signals, which are used by many species to communicate, contain components that can be analysed by multiple sensory channels (Otovic & Partan, 2009). Fish communicate through visual, chemical and acoustic signals often operating simultaneously to improve the chances of mating success, by indicating the physical quality or the motivation of the emitter (e.g. Amorim et al., 2013; Heuschele et al., 2009; Levine et al., 1980; Liley, 1982). It has been suggested that the acoustic modality is highly advantageous for territorial species, in which the nest site is frequently hidden, and the male is out of sight from the prospective mate (Myrberg Jr., 1981).

Another interesting finding from the current study is that females entered the male's territory, particularly the nest hollow, more frequently when accompanied by sound production than when the males were silent. Other studies suggest that different acoustic traits or morphological features could advertise male quality (genetic or phenotypic), serving as honest signals of different aspects of male quality in fish or sand gobies in particular (Amorim et al., 2013; Knapp & Kovach, 1991). According to Amorim et al. (2013), successful breeding *P. pictus* males produced more sounds and with a higher number of pulses than unsuccessful males.

### 4.3 Insights from the anatomical findings

Our findings indicate there are anatomical similarities in the musculoskeletal system of the pectoral girdle between the previously studied *Pomatoschistus* gobies and *O. croaticus* (Adriaens et al., 1993; Parmentier et al., 2017). This study provided the first anatomical dissections and μCT scans of the *O. croaticus* pectoral girdle and neurocranium. It is hypothesised that the Bauplan of soniferous gobies does not show deep significant modifications, meaning that the anatomy of soniferous species appears to be comparable to that of their silent relatives (Parmentier & Fine, 2016). To investigate the anatomy of the sound‐producing mechanism in gobies, Parmentier et al. (2013, 2017) undertook two empirical studies in two European gobies, gobiid *Gobius paganellus* (Gobiidae) and sand goby *P. pictus* (Gobionellidae), with the goal of testing the hypothesis of contraction of the pectoral girdle muscles. These multidisciplinary studies suggested strong similarities between the two gobies, and that sounds might be generated by the contraction of the *levator pectoralis* muscle. These results suggested that the pectoral girdle is likely involved in sound production. It is worth noting that sound production was coupled with nodding in *G. paganellus* or with lateral head movements in *P. pictus* (Parmentier et al., 2013, 2017). However, this does not indicate that head movements are exclusively responsible for the sound production. In this study, the pectoral girdle of *O. croaticus* consists of three functional osseous parts, with main elements present as in other dissected sand gobies (Adriaens et al., 1993; Parmentier et al., 2013, 2017). In addition, the *levator pectoralis* muscles, divided into two bundles (*pars lateralis* and *pars medialis*), were also found in *O. croaticus*, originating on the neurocranium and inserting onto the pectoral girdle. Four large radial bones were also present, forming the shoulder plate in *O. croaticus*. Lastly, the males performed lateral head movements or head elevation during sound emission. Although our study did not include methodologies such as muscle histology, high‐speed video or electromyography to fully corroborate the findings from earlier research (i.e. comprehensive description of the genuine sound generation mechanism), we believe there is sufficient evidence to hypothesise that the putative sound‐producing mechanism in *O. croaticus* could be related with the contractions of the *levator pectoralis* (*pars lateralis and medialis*) muscles and the pectoral girdle. Our assumptions are based on: (1) the observed anatomical similarities (i.e. muscle organisation and osseous structures) between *O. croaticus* and other tested sand gobies and (2) head (lateral) movements observed during sound emission. However, the detailed description of the sound‐producing mechanism in gobies is still expected and until then, the mechanism remains unidentified. Interestingly, in some situations, males were observed to perform body movements (lateral movements, head uplift, erection of fins), but without sound production, indicating that sound production requires more than just body movements. This supports the hypothesis that sounds are intentional and not only a by‐product of other activities such as breathing, feeding or swimming.

### 4.4 Acoustic difference between soniferous sand gobies

Freshwater sand gobies are considered important indicators for the conservation of Mediterranean inland aquatic ecosystems due to their wide range of habitats and high level of endemism (Vanhove et al., 2016). However, sand gobies are highly similar morphologically (Kovačić, 2008) and frequently live in sympatry (Miller, 1986), making their discrimination difficult. Several discrimination techniques have previously been proposed for gobioids, such as mitochondrial/nuclear DNA markers (Agorreta et al., 2013; Thacker et al., 2019; Vanhove et al., 2012), otoliths in the inner ear (Lombarte et al., 2018) and behaviour (Malavasi et al., 2012). Recently, the sounds (and their acoustic features) have become a useful parameter in determining the phylogenetic relationships in fish (Bolgan et al., 2020; Melotte et al., 2016; Parmentier et al., 2009; Rice & Bass, 2009), particularly in gobies (Horvatić et al., 2021; Malavasi et al., 2008). The aim of this study was not to infer the phylogenetic relationships between sand gobies, but rather to investigate how the species can be separated according to their acoustic features, and how well the sounds can be classified for each taxon. In the present study, we found interspecific differences among the sand gobies species based on acoustic properties. The LDA assigned each sound produced by sand gobies to the correct species with a discrimination rate of 86%. *Ninnigobius canestrinii* and *K. panizzae*, along with *P. pictus* and *P. marmoratus* (Italian population), were the species most separated from the other taxa on the LDS bi‐plot. Some authors have opposed the taxonomic separation of *O. croaticus* and *O. punctatissimus* into the genus *Orsinigobius*, and the isolation of *N. canestrinii* from the genus *Pomatoschistus* (Tougard et al., 2021). On the LDS bi‐plot, the two *Orsinigobius* taxa were closely situated, even though not forming one cluster. Furthermore, *P. minutus* from our study was in close proximity of the two *Orsinigobius* taxa. Interestingly, the ellipses of the two populations of *P. marmoratus* partially overlapped in the LDA, despite the fact they encompass individuals from a wide geographic area (the Po River delta in Italy and Parede/Arrábida in Portugal). However, the Italian population appeared partially isolated from the rest of the species.

Identifying a species can be a crucial discriminating challenge in the context of reproduction for related species living in sympatry, such as sand gobies. Acoustic signals, among others, might encrypt species affinity (Zeyl et al., 2016). Even though there are certain similarities between the sounds produced by soniferous sand gobies (such as their pulsatile nature, low‐frequency spectrum and low PRR; Figure 5), they were here successfully linked to the exact species, and these species were mutually separated. Therefore, from an evolutionary standpoint, our findings suggest that acoustic properties contain a certain amount of phylogenetic information, which is responsible for the interspecific divergence of the species from the present study. The observed acoustic variability may be employed to promote reproductive isolation or species recognition (Amorim, 2006; Horvatić et al., 2021). When applying the reduced dataset, the classification rate in LDA decreased from 86% to 69%, which is a less acceptable outcome, and it implies that interspecific discrimination becomes more difficult without certain (temporal) acoustic features, such as temperature‐dependent DUR and PRR in our case. Indeed, PRR is known to differ between closely related species, recorded at the same temperature (Lobel, 2001; Myrberg et al., 1978). Finally, the only remaining soniferous sand goby, which was previously acoustically investigated but was not included in this study, is the two‐spotted goby *Pomastoschistus flavescens* (Fabricius 1779; de Jong et al., 2016, 2017). During courting encounters, this species produces two different sound types (drums/pulsatile sounds and thumps), and it would be interesting to investigate how these sounds might combine with the acoustic signals of sand gobies from the current study in the future.

## 5 CONCLUSION

Our study demonstrates that the threatened and geographically restricted freshwater sand goby, *O. croaticus*, produces pulsatile sounds during intersexual laboratory experiments. The sounds were produced during courtship, but mainly pre‐spawning (and spawning) phases of the reproduction interactions with females. In addition, our results provide insight into the anatomy of the pectoral girdle (with the *levator pectoralis* muscles) which could be responsible for pulse emission. Finally, at the interspecific level, acoustic signals produced by soniferous sand gobies appear to be sufficiently different and species‐specific to enable the discrimination of species.

## AUTHOR CONTRIBUTIONS

**Sven Horvatić:** Conceptualization (lead); data curation (lead); formal analysis (equal); investigation (equal); methodology (equal); software (lead); visualization (lead); writing – original draft (lead); writing – review and editing (equal). **Eric Parmentier:** Formal analysis (equal); investigation (equal); methodology (equal); software (equal); visualization (equal); writing – original draft (equal); writing – review and editing (equal). **Stefano Malavasi:** Conceptualization (equal); data curation (equal); supervision (equal); writing – original draft (equal); writing – review and editing (equal). **Maria P. Clara Amorim:** Conceptualization (equal); data curation (equal); writing – original draft (equal); writing – review and editing (equal). **Paulo J. Fonseca:** Conceptualization (equal); data curation (equal); writing – original draft (equal); writing – review and editing (equal). **Davor Zanella:** Data curation (equal); resources (lead); supervision (equal); writing – original draft (equal); writing – review and editing (equal).

## FUNDING INFORMATION

This study was supported by the Ministry of Agriculture, Croatia.

## CONFLICT OF INTEREST STATEMENT

The authors declare that they have no competing interests.

## Supporting information


Figure S1

Figure S2

Tables S1‐S2

## Data Availability

Audio files and Excel tables are available from the Figshare data repository (https://doi.org/10.6084/m9.figshare.22787093.v1 and https://doi.org/10.6084/m9.figshare.22786952.v1).
