# Supplementary figures and images for: Endemic fish calling: Acoustics and reproductive behaviour of the Neretva dwarf goby Orsinigobius croaticus
Source: Ecol Evol. 2023 Nov 16;13(11):e10673. doi: 10.1002/ece3.10673 (PMC10654559; doi:10.1002/ece3.10673)

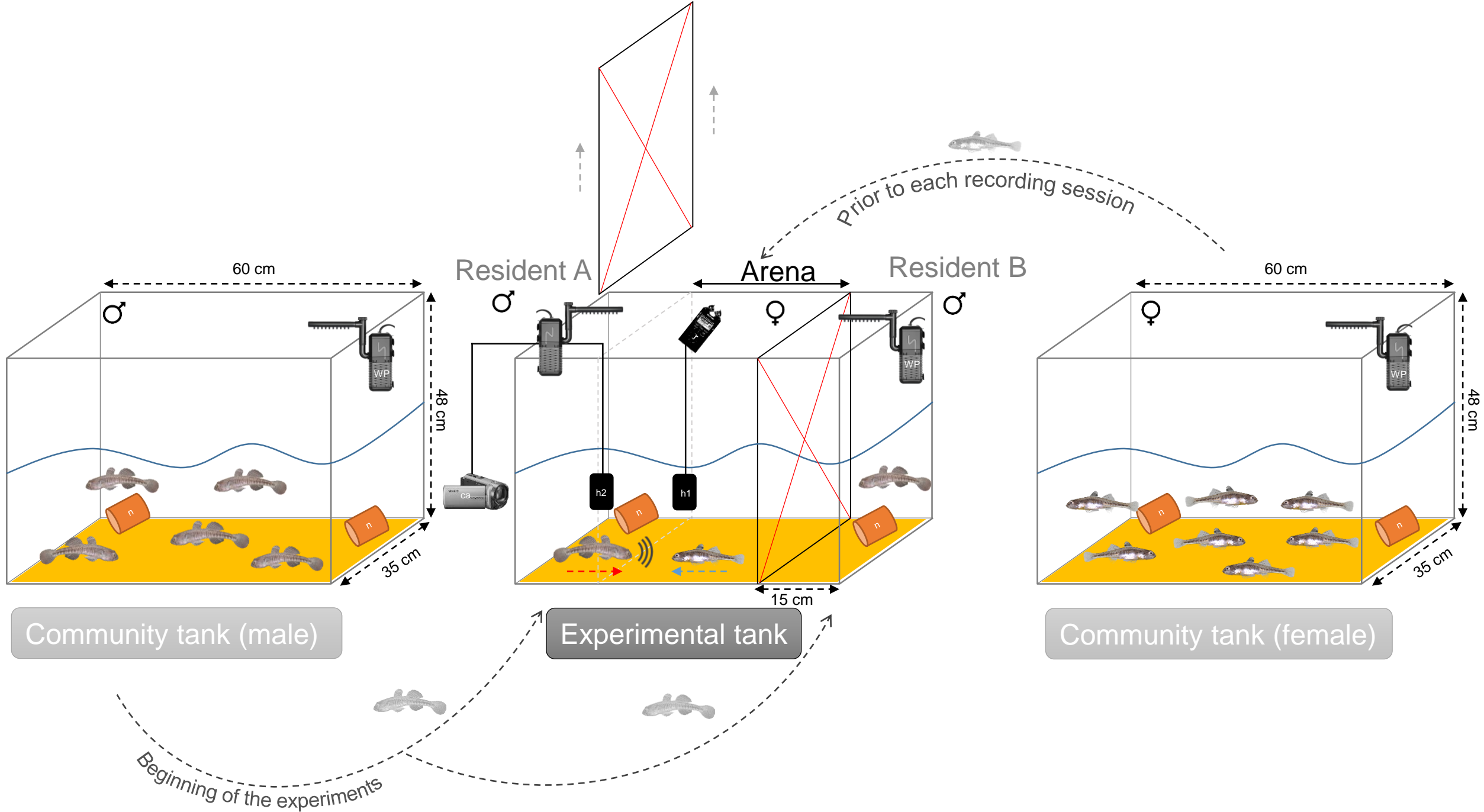

Supplement: Supplementary file 1 — Figure S1 [file ECE3-13-e10673-s001.pdf]

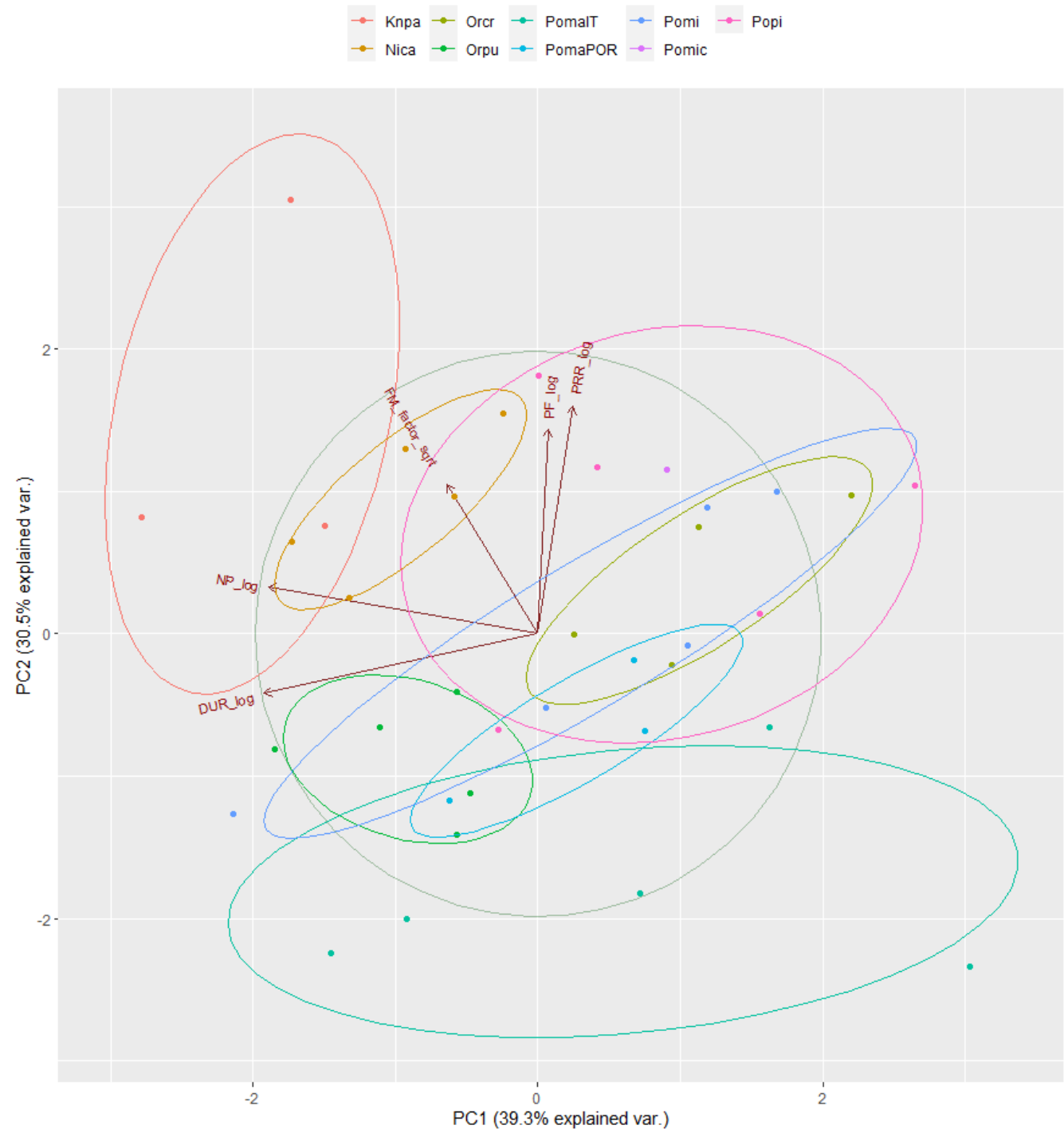

Supplement: Supplementary file 2 — Figure S2 [file ECE3-13-e10673-s003.pdf]
