# Supplementary material for: Endemic fish calling: Acoustics and reproductive behaviour of the Neretva dwarf goby Orsinigobius croaticus
Source: Ecol Evol. 2023 Nov 16;13(11):e10673. doi: 10.1002/ece3.10673 (PMC10654559; doi:10.1002/ece3.10673)
Supplement: Supplementary file 3 — Tables S1‐S2 [file ECE3-13-e10673-s002.docx]

**Endemic fish calling: acoustics and reproductive behaviour of the Neretva dwarf goby *Orsinigobius croaticus***

Horvatić Sven, Parmentier Eric, Malavasi Stefano, Amorim Maria P. Clara, Fonseca J. Paulo, Zanella Davor

**Table S1.** Explanations for the nine behaviour categories identified in male *Orsinigobius croaticus*. All nine behavioural categories are expressed during intersexual encounters with females.

| **Category** | **Description** |
| --- | --- |
| Ld | The male swims towards the female before quickly returning to the nest. This is followed by the dorsal and caudal fins fluttering (undulating). |
| Chs | The male chases the female on short (less than five cm) or long (more than five cm) distances away the nest, occasionally with an arched body and elevated dorsal fins. |
| App | The male approaches the female with elevated dorsal fins, while exhibiting “enlarged body” display and having dark head and fins areas. Occasionally, fluttering of male’s pectoral fins is present. This behaviour is frequently followed by Ld. |
| Crl | Once departing the nest, the male circles the female while lowering his dorsal fins and pointing his head downward. The behaviour and App can come concurrently sometimes. |
| Ndis | The male exposes the front half of the body outside the nest, while spreading the pectoral/dorsal fins and elevating the head. Male performs this display alone in the nest, or during female residence. |
| Fdis | The male exits the nest for the whole length of his body, or typically only half his body size, confronting the female frontally with his mouth closed, head erect, and opercula spread. This behaviour occasionally occurs when both sexes are in the nest. Male periodically undulates the pectoral fins. |
| Nrubb | During the female’s residence in the nest but before she engages in upside-down behaviour (oviposition), the male circles the nest ceiling by moving in zigzag patterns. |
| Pr-m | This behaviour includes male behaviours such as fins and body undulations (shivering), head and fin erection, parallel or opposite position to the female, abduction or adduction of the pectoral fins, head nudges, and body rubs in parallel position, that start as soon as the female enters the nest and before she displays her upside-down behaviour (oviposition). This behaviour occurs intermittently with Ndis and Fdis. The male’s colouration either totally or partially darkens (head and fins). |
| Spw | This behaviour starts when the female assumes the oviposition, i.e. when she starts with the upside-down display. The male circulates the nest ceiling by performing zig-zag movements. It stops once when the female leaves the nest. |

Ld - Lead, Chs - Chase, App - Approach and Crl - Circling (here considered courtship phase) were performed by the male outside the nest, while Ndis - Nest display, Fdis - Frontal display, Nrubb - Nest rubbing, Pr-m - Pre-mating (all considered as pre-spawning phases) were performed by the male within the nest. Spw - Spawning (i.e. spawning phase) was performed by the male within the nest.

**Table S2.** Descriptive statistics of the soniferous sand gobies acoustic characteristics. The mean (*x̄*) and standard deviation (*s.d.*) for each parameter were provided.

| **Species** | **DUR (ms)** | | **NP** | | **PRR (Hz)** | | **PF (Hz)** | | **FM (Hz)** | |
| --- | --- | --- | --- | --- | --- | --- | --- | --- | --- | --- |
|  | *x̄* | *s.d.* | *x̄* | *s.d.* | *x̄* | *s.d.* | *x̄* | *s.d.* | *x̄* | *s.d.* |
| *Ninnigobius canestrinii* | 752,5 | 140,0 | 25,4 | 3,5 | 33,9 | 3,3 | 153,5 | 32,3 | 2,4 | 2,1 |
| *Orsinigobius punctatissimus* | 856,3 | 157,2 | 23,0 | 3,6 | 27,1 | 2,6 | 127,6 | 16,1 | -3,2 | 1,0 |
| *Orsinigobius croaticus* | 442,1 | 90,3 | 14,3 | 3,1 | 32,5 | 2,4 | 137,4 | 41,0 | 0,9 | 0,0 |
| *Pomatoschisus microps* | 493,5 | / | 15,3 | / | 31,3 | / | 211,0 | / | -2,4 | / |
| *Pomatoschisus pictus* | 512,3 | 153,3 | 19,6 | 6,5 | 39,4 | 3,5 | 151,5 | 38,4 | -6,9 | 1,3 |
| *Pomatoschisus minutus* | 621,2 | 347,0 | 17,9 | 7,3 | 31,3 | 5,2 | 134,1 | 17,1 | -1,1 | 1,9 |
| *Pomatoschisus marmoratus* (ITA) | 693,8 | 313,8 | 17,2 | 6,6 | 25,9 | 1,8 | 125,0 | 22,7 | -8,6 | 2,7 |
| *Pomatoschisus marmoratus* (POR) | 618,0 | 125,1 | 17,8 | 4,4 | 28,9 | 2,5 | 131,2 | 40,2 | -4,4 | 0,7 |
| *Knipowitschia panizzae* | 1018,8 | 243,0 | 36,9 | 5,8 | 37,6 | 9,6 | 187,0 | 32,1 | -0,9 | 2,9 |

The statistics encompass 36 individuals of eight soniferous sand gobies (*P. marmoratus* divided onto two populations, ITA - Italian and POR - Portuguese), including *O. croaticus* and min - max: 3 - 5 individuals each with at least three sounds recorded, with the exception of a single sound from *P. microps*
